# Is Dentistry a New Profession?

**Published:** 1899-09

**Authors:** F. S. Casper


### Dental Department.

### Is Dentistry a New Profession?

F. S. CASPER, D. D. S.

It seems to be the general impression, not only with the laity, but with medical men and dentists themselves, that dentistry is a new profession as compared with medicine. The rapid strides this noble calling has made within the past fifty years, is no doubt in a measure, responsible for this opinion; for today we can face the world with the claim that we have, strictly speaking, a legitimate calling, a profession, hand in hand, among the great family of professions requiring years of study and training.

It is true that we have no knowledge of the art of dentistry being as old as the healing art; indeed, the history of our profession does not speak of it as one of the old professions, but Reginald Stuart Poole, in one of his lectures before the Royal Institutions said, "The physicians of Egypt during the Saite period, divided into specialists, as aurists, oculists, dentists and others for diseases of different parts of the body; and that the teeth of mummies show the excellence of the dental practice which had discovered the art of stopping with gold." Therefore, instead of the specialty of dentistry being new, it is as old as the medical practice of Egypt. That it has become, and is now recognized as eminently worthy of being studied and practiced as a specialty has been acknowledged by the foremost medical practitioners in this country and Europe.

Within the last twenty-eight years the College of Surgeons in London, realizing the necessity of a special education for the practice of dentistry, established a curriculum of study, on the completion of which the student is entitled to a degree; and by appropriate laws being passed no person can practice unless duly authorized. The apparent increase of the decay of the human teeth and diseases of the mouth justifies the necessity of our specialty, as a distinct pursuit, almost more than that of the aurist or oculist, because such diseases are more numerous than those of the eye and ear.

We therefore reiterate that the profession of the dentist is no longer in its infancy, but it is an old established calling, and we venture the assertion that our medical half-brothers will ere long arrive at the conclusion that it is a necessary adjunct to the medical branch of the State University, and that they will lend their aid toward this end. If this is done, as it surely will be, it will strengthen the medical branch beyond peradventure.

